# Sex differences in long-term outcomes among acute ischemic stroke patients with diabetes in China

**DOI:** 10.1186/s13293-015-0045-7

**Published:** 2015-12-04

**Authors:** Wenjuan Zhao, Zhongping An, Yan Hong, Guanen Zhou, Bin liu, Jingjing Guo, Yuanju Yang, Xianjia Ning, Jinghua Wang

**Affiliations:** Department of Neurology, Tianjin Huanhu Hospital, 122 Qixiangtai Road, Hexi District, 300060 Tianjin China; Tianjin Key Laboratory of Cerebral Vascular and Neurodegenerative Disease, 122 Qixiangtai Road, Hexi District, 300060 Tianjin China; Department of Ultrasound, Tianjin Huanhu Hospital, 122 Qixiangtai Road, Hexi District, 300060 Tianjin China; Department of Radiology, Tianjin Huanhu Hospital, 122 Qixiangtai Road, Hexi District, 300060 Tianjin China; Department of Epidemiology, Tianjin Neurological Institute, Tianjin Medical University General Hospital, Tianjin, China; Department of Neurology, Tianjin Medical University General Hospital, Tianjin, China

**Keywords:** Sex differences, Acute ischemic stroke, Diabetes mellitus, Outcome, Risk factors

## Abstract

**Background:**

Diabetes has been shown to be significantly associated with poor outcome after stroke. However, the sex differences in stroke outcome among patients with diabetes are unknown. Therefore, we aimed to assess the sex differences in long-term prognosis among acute ischemic stroke patients with diabetes.

**Methods:**

The ischemic stroke patients with diabetes were recruited to this study between May 2005 and September 2014 in Tianjin, China. Sex differences in mortality, dependency (modified rank scale > 2), and recurrence at 3, 12, and 36 months after stroke were analyzed.

**Results:**

A total of 2360 patients were recruited in this study. The age of stroke onset, National Institute of Health stroke scale (NIHSS), and modified rank scale (mRS) on admission were greater in women than in men (*P* < 0.05). Women were more likely to have hypertension, obesity, atrial fibrillation, and dyslipidemias. In contrast, men were more likely to have artery stenosis, current smoking, and alcohol consumption (*P* < 0.001). There was higher mortality in women than in men at 3 months (7.9 % vs 5.2 %), 12 months (12.2 % vs 8.2 %), and 36 months (21.9 % vs 16.1 %) after stroke; but no differences were found in dependency and recurrence. Sex differences were found in associated factors of outcomes by time-point. Trial of Org 10172 in Acute Stroke Treatment (TOAST) of large artery atherothrombosis (LAA), cardioembolism (CE), and smoking were risk factors of outcomes in women at short term and medium term; but atrial fibrillation (AF), obesity, and alcohol were risk factors of outcomes in men at medium term and long term.

**Conclusions:**

These findings suggest that it is crucial to establish the individual scheme of therapy for every patient by different risk factors of stroke, strengthen the rehabilitation of stroke, and carry on the health education early for the secondary prevention of stroke in patients with diabetes mellitus (DM).

## Background

Although age-standardized rates of stroke mortality have decreased worldwide in the past two decades, the absolute numbers of annual stroke cases, stroke survivors, related deaths, and the global burden of stroke disability-adjusted life-years are great and continue to increase. A recent study demonstrated that stroke is the second most common cause of death and the third most common cause of disability-adjusted life-years worldwide in 2010 [1, 2]. Moreover, reports from 2010 indicate that stroke is the second cause of death in China [3].

The prevalence of diabetes mellitus (DM) is rapidly increasing worldwide, and DM is predicted to become the seventh leading cause of death in the world by 2030 [4]. According to a report by the International Diabetes Federation, the prevalence of diabetes in 2011 was approximately 8.3 % [5]. In addition, about half of the worldwide diabetic population resides in Asian countries, particularly in China and India, where diabetes is a major public health burden [5].

It is well known that diabetes is significantly associated with stroke, and patients with diabetes are at greater risk of stroke than individuals without diabetes [6–9].

There is growing recognition of the clinical and public health importance of stroke in women. Although age-specific stroke incidence and mortality rates are higher in men than in women, stroke affects a greater number of women because of their increased longevity. A previous study demonstrated that there was a significant increase in the incidence of first-ever stroke in women annually and a declining trend in the male/female rate ratio in rural China over the past 21 years [8]. Moreover, stroke-related outcomes, including disability and quality of life, are consistently worse in women than in men [9]. A few studies have also reported the association between diabetes and long-term prognosis of patients following acute ischemic stroke (AIS) [10, 11]. However, the sex differences in clinical profiles and prognosis among AIS patients with DM still remains unclear.

Therefore, we aimed to assess sex differences in stroke subtypes, severity, pre-stroke risk factors, and impact of DM on short-term (3 months), medium-term (12 months), and long-term (36 months) outcome after stroke.

## Methods

### Patients

All consecutive patients with first-ever AIS who were admitted to the Stroke Unit in Tianjin Huanhu Hospital within 72 h of stroke onset between May, 2005, and September, 2014, were recruited to this study. Clinical diagnosis of stroke was made according to the World Health Organization criteria and was confirmed by neuroimaging (including CT or MRI) [12]. Cases of transient ischemic attack were excluded from this study. Patients with a premorbid modified rank scale (mRS) > 2 were not assessed in this study. DM was defined by a self-reported previous medical history of DM or the use of anti-diabetic medicine.

The detailed information on ischemic stroke subtype, stroke severity, previous medical history, stroke risk factors, laboratory examination, and prognosis at 3, 12, and 36 months after stroke was collected.

A standardized questionnaire was administered at 3, 12, and 36 months after stroke to collect detailed information on ischemic stroke subtypes, stroke severity, previous medical history, stroke risk factors, laboratory examination, and prognosis.

### Ethics, consent, and permissions

The study was approved by the ethics committee for medical research at Tianjin Huanhu Hospital and the Tianjin Health Bureau, and a written informed consent for each participant was obtained during recruitment.

### Definition

Stroke subtypes were defined according to the Trial of Org 10172 in Acute Stroke Treatment (TOAST) classification criteria, which included large artery atherothrombosis (LAA), cardioembolism (CE), small artery occlusion (SAO), other causes, and undetermined [13].

Neurological function deficits were described using the National Institute of Health stroke scale (NIHSS), Bethel index (BI), and mRS on admission. Stroke severity was categorized into three groups on the basis of NIHSS score: mild, ≤7; moderate, 8–16; and severe, ≥17 [14].

Stroke risk factors included a medical history of hypertension (defined as self-reported history of hypertension or using antihypertension drugs), diabetes mellitus (DM, defined as history of DM or using hypoglycemic medications at discharge), dyslipidemias (defined as self-reported history of all types of dyslipidemia or oral antidyslipidemia drugs, or using antidyslipidemia drugs at discharge), atrial fibrillation (AF, defined as history of AF, confirmed by at least one electrocardiogram or the presence of the arrhythmia during hospitalization), and modifiable lifestyle factors, including current smoking status, alcohol consumption, and obesity (body mass index ≥30 kg/m^2^).

### Outcomes

Stroke outcomes were described by mortality, recurrence, and dependency rates at 3, 12, and 36 months after stroke; outcomes were assessed by face-to-face or telephone follow-up. Death was defined as all-cause cumulative death at the corresponding time-points after stroke, and this information was collected from patients’ family members or telephone follow-up. Recurrence was defined as all new-onset vascular events (stroke and myocardial infarction) occurring within 30 days after initial stroke. Dependency was defined as mRS > 2 [15]. The mortality rate was calculated as the proportion of death among all patients over the same period after stroke. The recurrence rate was the proportion of patients with recurrence among all surviving patients followed up by face-to-face interview or telephone call, and the dependency rate was the proportion of patients with mRS > 2 among all survivors who were followed up by face-to-face interview.

### Statistical analysis

Continuous variable was presented as means (SD) or median and range were compared between men and women using the Student’s *t* test or Mann–Whitney *U* test. Dichotomous variable was presented as numbers (percentages) and compared using the chi-square test. Sex differences in outcomes were assessed by logistic regression models and presented as relative risk (RR) with 95 % confidence intervals (CIs). The dependent variable was outcome at 3, 12, and 36 months after stroke and was defined as “yes” or “no”; the independent variables included age (defined as a continuous variable), TOAST classification (defined as a categorical variable with small artery occlusion as reference), stroke severity (defined as a categorical variable with mild stroke as reference), and previous medical histories of hypertension, AF, dyslipidemia, artery stenosis, obesity, current smoking status, and alcohol consumption (defined as dichotomous “yes” or “no” variables). The multivariate analysis was performed using age, TOAST classification, stroke severity, hypertension, AF, dyslipidemia, artery stenosis, obesity, current smoking status, and alcohol consumption as the covariates. All statistical analyses were performed using SPSS version 15.0 (SPSS Inc., Chicago, IL), and two-tailed *P* values <0.05 were considered statistically significant.

## Results

**Fig. 1:**
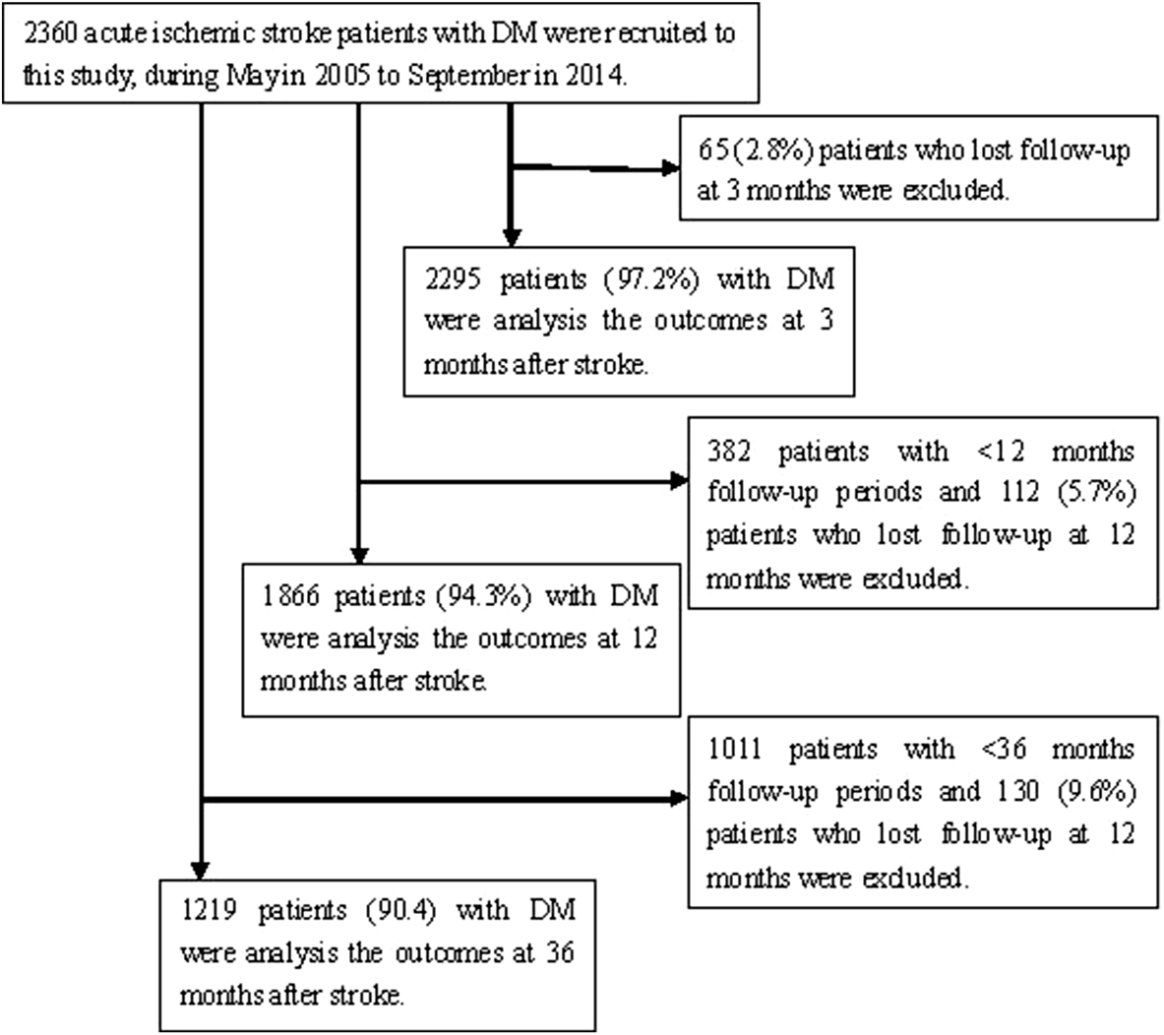
Flow diagram of participants

A total of 7565 AIS patients were recruited in this study during study periods; of these patients, 2360 (31.2 %) AIS patients with DM were registered, including 1450 (28.9 %) men and 910 (35.6 %) women. The percentages of patients who completed follow-up at 3, 12, and 36 months after stroke were 97.2, 94.3, and 90.4 %, respectively (Fig. 1).

As shown in Table 1, the age of patients at the time of AIS was greater in women than in men (mean age of 66.4 years in women vs. 62.7 years in men; *P* = 0.004). More CE occurred in women than in men (5.6 % vs 2.6 %), and compared to men, more women experienced moderate and severe stroke (40.4 % vs 34.0 %; *P* = 0.001). Moreover, the NIHSS, BI, and mRS on admission were greater in women than in men (*P* < 0.001).

**Table 1:** The sex differences in clinical characteristics of acute ischemic stroke with diabetes on admission

Characteristics	Men	Women	*P*
Numbers, *n* (%):	1450 (61.4)	910 (38.6)	—
Age, year, means (SE)	62.7 (11.1)	66.4 (9.8)	<0.001
TOAST classification, *n* (%):			0.004
Large artery atherothrombosis	1054 (72.7)	634 (69.7)	
Cardiac embolism	37 (2.6)	51 (5.6)	
Small artery occlusion	315 (21.7)	190 (13.1)	
Other determined etiology	17 (1.2)	10 (1.1)	
Undetermined etiology	19 (1.3)	15 (1.6)	
Stroke severity, *n* (%):			0.003
Mild	957 (66.0)	542 (59.6)	
Moderate	376 (26.9)	267 (29.3)	
Severe	115 (8.0)	101 (11.1)	
Neurological function deficit: means (SE)			
NIHSS	5 (0–36)	6 (0–35)	<0.001
BI	60 (0–100)	55 (0–100)	<0.001
mRS	3 (0–6)	4 (0–5)	<0.001

*TOAST* trial of Org 10172 in acute stroke treatment, *NIHSS* National Institute of Health stroke scale, *BI* bethel index, *mRS* modified rank scale

The prevalence rates of hypertension, obesity, AF, and dyslipidemias were significantly higher in women than in men (82.9 % vs 74.8 %, 24.5 % vs 11.9 %, 8.6 % vs 5.2 %, and 41.9 % vs 36.1 %, respectively; *P* < 0.05). In contrast, men were more likely than women to have artery stenosis, current smoking status, and alcohol consumption, with corresponding prevalence rates of 29.4 % vs 23.7 %, 45.5 vs 12.0 %, and 23.9 % vs 0.8 %, respectively (*P* < 0.05, Table 2).

**Table 2:** The sex differences in risk factors of acute ischemic stroke in patients with diabetes

Risk factors	Men	Women	*P*
Hypertension, *n* (%)	1084 (74.8)	754 (82.9)	<0.001
Obesity, *n* (%)	173 (11.9)	223 (24.5)	<0.001
Atrial fibrillation, *n* (%)	76 (5.2)	78 (8.6)	0.001
Dyslipidemias, *n* (%)	524 (36.1)	381 (41.9)	0.005
Artery stenosis, *n* (%)	427 (29.4)	216 (23.7)	0.002
Current smoking, *n* (%)	660 (45.5)	109 (12.0)	<0.001
Alcohol consumption, *n* (%)	347 (23.9)	7 (0.8)	<0.001

Table 3 shows that the mortality rates in women were significantly higher than in men at 3 months (7.9 % vs 5.2 %), 12 months (12.2 % vs 8.2 %), and 36 months (21.9 % vs 16.1 %) after stroke (*P* < 0.05); with the RR (95 % CI) of 1.56 (1.11, 2.19), 1.55 (1.15, 2.09), and 1.46 (1.11, 1.93). But there were not sex differences in dependency and recurrence rate at all time-points. After adjusting for age, TOAST classification, stroke severity, and other risk factors, the multivariate regression analysis showed that sex was not an independent predictor of death after stroke; the RRs (95 % CIs) were 1.10 (0.74, 1.63; *P* = 0.646) 3 months after stroke, 1.07 (0.75, 1.53; *P* = 0.710) 12 months after stroke, and 1.08 (0.76, 1.51; *P* = 0.680) 36 months after stroke (Table 4). The age and severity of stroke were risk factors of outcome in AIS patients with DM across sex and time-point. At 3 months, there was a higher risk of death in women with TOAST classification of LAA, with an RR (95 % CI) of 6.26 (1.48, 26.5); however, the risk of dependency decreased by 62 % in obese men, with an RR (95 % CI) of 0.38 (0.17, 0.83). At 12 months after stroke onset, AF increased the risk of death in men, with an RR (95 % CI) of 3.30 (1.25, 8.72). The risk factors in women were LAA and CE for death, CE and smoking for recurrence, and smoking for dependency; the corresponding RRs (95 % CIs) were 3.03 (1.24, 7.43), 4.97 (1.25, 19.8), 2.84 (1.03, 7.80), 1.76 (1.03, 3.02), and 1.81 (1.05, 3.12), respectively. At 36 months in men, LAA and AF increased the risk of death, obesity and alcohol increased the risk of recurrence, and alcohol use increased the risk of dependency; the corresponding RRs (95 % CIs) were 2.25 (1.18, 4.29), 3.51 (1.29, 9.56), 1.68 (1.03, 2.74), 1.55 (1.03, 2.33), and 1.66 (1.09, 2.54), respectively. In women, LAA was a risk factor of death, with an RR (95 % CI) of 2.25 (1.10, 4.59) (Tables 5 and 6).

**Table 3:** The sex differences in outcome among acute ischemic stroke patients with diabetes

Outcomes	Men	Women	OR (95 % CI)	*P*
3 months:				
Mortality	74 (5.2)	70 (7.9)	1.56 (1.11, 2.19)	0.009
Dependency	138 (10.3)	85 (10.4)	1.02 (0.76, 1.35)	0.920
Recurrence	136 (10.2)	84 (10.3)	1.02 (0.76, 1.36)	0.904
12 months:				
Mortality	100 (8.2)	93 (12.2)	1.55 (1.15, 2.09)	0.004
Dependency	315 (28.2)	184 (27.5)	0.97 (0.83, 1.26)	0.742
Recurrence	330 (28.8)	203 (29.2)	1.02 (0.82, 1.23)	0.850
36 months:				
Mortality	133 (16.1)	115 (21.9)	1.46 (1.11, 1.93)	0.007
Dependency	371 (53.6)	216 (53.2)	0.97 (0.76, 1.24)	0.797
Recurrence	414 (54.0)	254 (54.4)	1.02 (0.81, 1.28)	0.888

**Table 4:** The effected factors of outcomes (OR with 95 % CI)

Risk factors	Mortality			Recurrence			Dependency		
	3 months	12 months	36 months	3 months	12 months	36 months	3 months	12 months	36 months
Sex (male)	0.90 (0.60, 1.35)	0.94 (0.65, 1.36)	0.95 (0.67, 1.35)	1.05 (0.76, 1.45)	1.21 (0.95, 1.54)	1.09 (0.84, 1.43)	1.05 (0.76, 1.46)	1.26 (0.98, 1.61)	1.15 (0.87, 1.53)
Age	1.05 (1.03, 1.07)*	1.06 (1.04, 1.08)*	1.07 (1.05, 1.09)*	1.03 (1.01, 1.04)*	1.02 (1.01, 1.03)*	1.02 (1.01, 1.03)*	1.03 (1.01, 1.04)*	1.02 (1.01, 1.03)*	1.02 (1.01, 1.03)*
TOAST:									
LAA	2.28 (1.18, 4.40)*	2.08 (1.22, 3.56)*	2.27 (1.41, 3.65)*	1.25 (0.86, 1.82)	0.94 (0.73, 1.21)	1.01 (0.79, 1.33)	1.27 (0.87, 1.85)	0.97 (0.75, 1.26)	1.09 (0.82, 1.46)
CE	0.91 (0.30, 2.76)	1.73 (0.65, 4.57)	1.29 (0.48, 3.49)	1.58 (0.66, 3.79)	1.46 (0.72, 2.94)	1.43 (0.60, 3.35)	1.60 (0.67, 3.84)	1.31 (0.62, 2.75)	1.17 (0.42, 3.27)
Severity:									
Moderate	4.73 (2.92, 7.66)*	4.19 (2.80, 6.29)*	3.09 (2.15, 4.42)*	1.31 (0.95, 1.82)	1.74 (1.37, 2.20)*	1.30 (0.99, 1.70)	1.31 (0.95, 1.81)	1.73 (1.36, 2.21)*	1.33 (1.00, 1.77)*
Severe	16.75 (10.05, 27.91)*	13.54 (8.57, 21.38)*	10.30 (6.55, 16.19)*	2.16 (1.34, 3.47)*	3.02 (2.03, 4.49)*	1.82 (1.12, 2.97)*	2.12 (1.32, 3.41)*	2.79 (1.83, 4.24)*	1.72 (0.96, 3.08)
Hypertension	1.02 (0.64, 1.61)	1.09 (0.72, 1.64)	1.26 (0.86, 1.86)	0.98 (0.70, 1.39)	1.03 (0.80, 1.33)	1.14 (0.86, 1.49)	0.97 (0.69, 1.37)	0.95 (0.74, 1.23)	1.05 (0.79, 1.40)
Smoking	1.36 (0.85, 2.17)	0.94 (0.60, 1.47)	0.92 (0.60, 1.40)	0.95 (0.65, 1.37)	0.98 (0.75, 1.28)	0.83 (0.62, 1.12)	0.93 (0.64, 1.34)	0.97 (0.73, 1.27)	0.80 (0.59, 1.10)
AF	2.13 (1.01, 4.48)*	1.46 (0.72, 2.99)	2.33 (1.07, 5.09)*	1.19 (0.60, 2.36)	1.08 (0.63, 1.86)	0.90 (0.45, 1.81)	1.17 (0.59, 2.31)	1.11 (0.63, 1.96)	1.36 (0.58, 3.23)
Dyslipidemia	1.02 (0.68, 1.51)	0.99 (0.69, 1.42)	0.82 (0.58, 1.16)	1.07 (0.79, 1.45)	1.18 (0.95, 1.48)	0.85 (0.67, 1.09)	1.04 (0.77, 1.41)	1.23 (0.98, 1.54)	0.86 (0.67, 1.11)
Artery stenosis	0.66 (0.41, 1.05)	0.73 (0.48, 1.11)	0.79 (0.54, 1.15)	1.08 (0.78, 1.50)	1.08 (0.85, 1.38)	1.18 (0.85, 1.44)	1.12 (0.81, 1.55)	1.08 (0.84, 1.38)	1.06 (0.80, 1.40)
Obesity	0.81 (0.48, 1.37)	0.71 (0.43, 1.17)	0.63 (0.38, 1.02)	0.51 (0.32, 0.82)	0.85 (0.63, 1.14)	1.56 (1.11, 2.19)*	0.50 (0.32, 0.80)	0.80 (0.59, 1.09)	1.52 (1.07, 2.16)*
Alcohol	0.41 (0.17, 0.96)*	0.66 (0.32, 1.37)	0.92 (0.49, 1.74)	1.04 (0.65, 1.68)	0.79 (0.55, 1.12)	1.49 (1.01, 2.20)*	1.03 (0.64, 1.66)	0.82 (0.57, 1.17)	1.61 (1.07, 2.42)*

*P < 0.05 for multivariable regression model

**Table 5:** The associated factors of outcomes in men (OR with 95 % CI)

Risk factors	Mortality			Recurrence			Dependency		
	3 months	12 months	36 months	3 months	12 months	36 months	3 months	12 months	36 months
Age	1.04 (1.02, 1.07)*	1.05 (1.03, 1.08)*	1.07 (1.05, 1.10)*	1.02 (1.00, 1.04)*	1.02 (1.00, 1.03)*	1.01 (0.99, 1.03)	1.02 (1.00, 1.04)*	1.02 (1.00, 1.03)*	1.02 (1.00, 1.03)*
TOAST:									
LAA	1.30 (0.6, 2.85)	1.54 (0.78, 3.04)	2.25 (1.18, 4.29)*	1.05 (0.66, 1.65)	0.93 (0.67, 1.28)	0.90 (0.63, 1.28)	1.06 (0.67, 1.68)	0.94 (0.68, 1.31)	0.95 (0.66, 1.37)
CE	0.14 (0.02, 1.00)	0.50 (0.12, 2.12)	0.65 (0.16, 2.68)	1.14 (0.29, 4.55)	0.59 (0.21, 1.65)	0.53 (0.17, 1.69)	1.15 (0.29, 4.55)	0.63 (0.21, 1.91)	0.61 (0.16, 2.32)
Severity:									
Moderate	5.93 (3.02, 11.6)*	5.04 (2.87, 8.83)*	2.72 (1.67, 4.43)*	1.35 (0.89, 2.05)	1.87 (1.38, 2.54)*	1.49 (1.05, 2.11)*	1.37 (0.90, 2.07)	1.83 (1.34, 2.50)*	1.51 (1.05, 2.20)*
Severe	24.0 (11.67, 49.4)*	16.72 (8.79, 31.8)*	11.3 (6.09, 20.9)*	2.40 (1.27, 4.52)*	3.54 (2.07, 6.07)*	2.32 (1.19, 4.53)*	2.37 (1.26, 4.46)*	3.32 (1.88, 5.86)*	2.87 (1.22, 6.78)*
Hypertension	0.97 (0.54, 1.75)	1.13 (0.67, 1.91)	1.52 (0.92, 2.53)	1.09 (0.72, 1.67)	1.00 (0.74, 1.35)	1.24 (0.89, 1.74)	1.07 (0.70, 1.62)	0.95 (0.70, 1.29)	1.17 (0.82, 1.66)
Smoking	1.26 (0.69, 2.31)	0.81 (0.46, 1.41)	0.86 (0.51, 1.43)	0.83 (0.54, 1.27)	0.78 (0.57, 1.07)	0.73 (0.52, 1.03)	0.81 (0.53, 1.24)	0.78 (0.57, 1.07)	0.72 (0.50, 1.03)
AF	2.80 (0.93, 8.40)	3.30 (1.25, 8.72)*	3.51 (1.29, 9.56)*	0.85 (0.30, 2.43)	1.48 (0.72, 3.07)	1.40 (0.56, 3.49)	0.84 (0.29, 2.37)	1.24 (0.58, 2.67)	1.51 (0.52, 4.41)
Dyslipidemia	0.89 (0.50, 1.58)	0.84 (0.50, 1.42)	0.86 (0.53, 1.37)	1.00 (0.67, 1.48)	1.26 (0.95, 1.68)	0.81 (0.60, 1.11)	0.97 (0.65, 1.43)	1.32 (0.98, 1.76)	0.81 (0.58, 1.13)
Artery stenosis	0.62 (0.32, 1.18)	0.57 (0.32, 1.02)	0.87 (0.53, 1.40)	1.02 (0.68, 1.54)	1.07 (0.80, 1.45)	1.20 (0.86, 1.66)	1.09 (0.73, 1.63)	1.08 (0.80, 1.46)	1.16 (0.82, 1.65)
Obesity	0.87 (0.35, 2.15)	0.67 (0.27, 1.65)	0.51 (0.22, 1.16)	0.38 (0.17, 1.15)	0.77 (0.50, 1.20)	1.68 (1.03, 2.74)*	0.38 (0.17, 0.83)*	0.75 (0.48, 1.18)	1.63 (0.99, 2.69)
Alcohol	0.43 (0.18, 1.04)	0.75 (0.35, 1.59)	1.00 (0.51, 1.95)	1.00 (0.65, 1.65)	0.86 (0.60, 1.24)	1.55 (1.03, 2.33)*	0.99 (0.60, 1.63)	0.88 (0.61, 1.28)	1.66 (1.09, 2.54)*

*P < 0.05 for multivariable regression model

**Table 6:** The associated factors of outcomes in women (OR with 95 % CI)

Risk factors	Mortality			Recurrence			Dependency		
	3 months	12 months	36 months	3 months	12 months	36 months	3 months	12 months	36 months
Age	1.05 (1.02, 1.09)*	1.07 (1.04, 1.11)*	1.06 (1.03, 1.10)^a^	1.04 (1.01, 1.07)*	1.04 (1.02, 1.06)*	1.03 (1.01, 1.05)*	1.04 (1.02, 1.07)*	1.03 (1.01, 1.05)*	1.03 (1.01, 1.06)*
TOAST:									
LAA	6.26 (1.48, 26.5)*	3.03 (1.24, 7.43)*	2.25 (1.10, 4.59)*	1.78 (0.90, 3.54)	0.92 (0.60, 1.41)	1.21 (0.77, 1.90)	1.82 (0.92, 3.62)	0.95 (0.61, 1.47)	1.41 (0.88, 2.26)
CE	4.49 (0.74, 27.2)	4.97 (1.25, 19.8)*	2.72 (0.64, 11.5)	2.36 (0.69, 8.10)	2.84 (1.03, 7.80)*	3.83 (0.92, 15.96)	2.40 (0.70, 8.20)	2.26 (0.79, 6.48)	2.33 (0.41, 13.2)
Severity:									
Moderate	3.79 (1.88, 7.63)*	3.54 (1.94, 6.47)*	3.84 (2.21, 6.66)*	1.20 (0.70, 2.04)	1.58 (1.07, 2.33)*	1.05 (0.68, 1.62)	1.17 (0.69, 1.98)	1.62 (1.09, 2.42)*	1.11 (0.69, 1.78)
Severe	11.7 (5.54, 24.6)*	12.2 (6.17, 24.1)*	9.98 (5.02, 19.8)*	1.90 (0.91, 3.97)	2.49 (1.36, 4.56)*	1.40 (0.67, 2.94)	1.84 (0.88, 3.85)	2.34 (1.22, 4.47)*	1.11 (0.48, 2.55)
Hypertension	1.06 (0.49, 2.29)	1.00 (0.59, 1.96)	1.01 (0.54, 1.88)	0.79 (0.43, 1.44)	1.06 (0.67, 1.67)	0.88 (0.55, 1.43)	0.80 (0.44, 1.47)	0.95 (0.60, 1.52)	0.79 (0.47, 1.32)
Smoking	1.61 (0.76, 3.43)	1.25 (0.59, 1.96)	1.13 (0.53, 2.41)	1.25 (0.62, 2.53)	1.76 (1.03, 3.02)*	1.13 (0.61, 2.11)	1.22 (0.60, 2.47)	1.81 (1.05, 3.12)*	1.13 (0.59, 2.15)
AF	1.87 (0.69, 5.05)	0.71 (0.25, 2.02)	1.32 (0.37, 4.68)	1.73 (0.68, 4.45)	0.94 (0.41, 2.14)	0.66 (0.20, 2.18)	1.72 (0.67, 4.39)	1.06 (0.46, 2.46)	1.12 (0.25, 4.95)
Dyslipidemia	1.10 (0.63, 1.94)	1.19 (0.71, 2.00)	0.79 (0.48, 1.30)	1.20 (0.73, 1.96)	1.06 (0.73, 1.52)	0.88 (0.59, 1.30)	1.17 (0.72, 1.91)	1.09 (0.75, 1.58)	0.94 (0.62, 1.42)
Artery stenosis	0.73 (0.37, 1.47)	0.99 (0.54, 1.84)	0.67 (0.37, 1.22)	0.24 (0.71, 2.16)	1.25 (0.82, 1.91)	1.06 (0.67, 1.69)	1.22 (0.70, 2.13)	1.10 (0.71, 1.71)	0.94 (0.58, 1.52)
Obesity	0.81 (0.42, 1.56)	0.74 (0.40, 1.37)	0.71 (0.38, 1.33)	0.63 (0.34, 1.15)	0.90 (0.59, 1.37)	1.42 (0.88, 2.28)	0.62 (0.34, 1.13)	0.85 (0.55, 1.31)	1.47 (0.89, 2.43)
Alcohol	—	—	—	4.07 (0.69, 24.1)	0.40 (0.04, 3.67)	—	4.05 (0.68, 24.0)	0.39 (0.04, 3.56)	—

*P < 0.05 for multivariable regression model

## Discussion

DM is an independent risk factor for ischemic stroke. Relative risk of ischemic stroke in patients with DM is between 1.8 and 6.0 [16]. Moreover, the prevalence of DM ranges from 21 to 44 % in patients with AIS [17, 18].

In this large stroke registry from a single center in China, we found that compared to men, women were more likely to be older; have previous medical histories of hypertension, obesity, AF, and dyslipidemias; and have higher levels of TC, TG, HDL-C, and LDL-C. Concurrently, women had worse outcomes than men. A younger age of stroke onset (with a mean age of 64.1 years overall) was found in this study compared to other studies, and this can be explained by the significantly increased incidence of stroke in people aged 20–64 years in low-income and middle-income countries in 1990–2010 [19].

We found a 31.2 % prevalence of DM overall, with prevalence rates of 28.9 % in men and 35.6 % in women; the prevalence of DM was significantly higher in women than in men. This rate is higher than that recently reported in The China National Stroke Registry, which the prevalence of DM was 26.99 % overall, 24.83 % in men and 30.46 % in women [20].

A large number of studies have reported that women tend to have stroke at an older age compared with men [21–23]. In accordance with these findings, women were 3.7 years older at stroke onset than men in this study. The neuroprotective effects of estradiol in women, which include hormones, sex chromosomes, or other sex-specific etiologies that could confer female resilience and result in a later age of onset [24, 25], may explain the older age of women at stroke onset.

Previous studies have indicated that there was a higher proportion of CE in women than in men [23, 26]. Consistent with these findings, we found that there was a higher proportion of CE in women than in men. Sex differences in stroke subtype are thought to be associated with the increased prevalence of AF in older women [27].

Several studies have reported that women were more likely to have severe stroke than men [22, 23], though this was not found in other studies [28, 29]. In the present study, we found a significantly increased frequency of moderate and severe stroke (NIHSS score >7) in women compared with men; NIHSS and mRS on admission were obviously greater in women than in men. These findings may be partly explained by the older age of women at stroke onset and prehospital delay [30].

Previous studies have indicated that the main vascular risk factors, including hypertension, DM, AF, dyslipidemia, and obesity, occur more often in women than in men; these risk factors have been recognized to increase the risk of ischemic stroke both in men and women [14, 31, 32]. AIS patients with DM were more likely to have hypertension, AF, dyslipidemias, and obesity [33, 34]. In contrast, AIS patients without DM were more likely to have CE and AF [20]. In this study, we found that female AIS patients were more likely to have hypertension, obesity, AF, and dyslipidemias. Women were less likely than men to achieve target values for controlled risk factors, which may explain the sex differences in risk factors among diabetic AIS patients [35]. A lower prevalence of AF in this stroke registry (8.4 % overall) than in other studies may be attributed to the lower risk of AF and a low rate of dynamic electrocardiogram and long time-interval electrocardiogram testing.

DM is associated with higher mortality [20, 36–38], dependency [39, 40], and recurrence rates after stroke [38–42]. Concurrently, a great deal of studies have observed a poor outcome in women [41, 42].

In this study, we found a significantly higher mortality rate in women than in men. Simultaneously, we found that age and severity of stroke were associated to stroke outcomes both in men and in women; LAA, CE, and smoking were the risk factors of outcomes in women at short term and medium term after stroke onset. In men, AF was the risk factor of death at medium term and long term after stroke onset; obesity was the protective factor of recurrence and dependency at short term after stroke onset, but the risk factor of recurrence at long term after stroke onset; and alcohol consumption was the risk factor of recurrence and dependency at long term after stroke onset. These may be explained partly by higher proportion of moderate and severe stroke (40.4 %) and CE in women and alcohol consumption in men.

Consistent with the previous studies, we found that there were no sex differences in outcomes among stroke patients with DM in this study. However, we assessed differences in the factors affecting stroke outcomes between diabetic men and women. We found that age and stroke severity were independent factors affecting stroke outcomes. Moreover, there were more factors in diabetic men than in diabetic women. We assessed the level of HbA1c on admission, but no difference was found between diabetic men and women, with a rate of HbA1c ≥ 6.5 % of 79.2 % in diabetic women and 78.0 % in diabetic men (*P* = 0.479). HbA1c was not found to affect stroke outcomes in either men or women with diabetes. Thus, the higher prevalence of traditional stroke risk factors, including artery stenosis, current smoking status, and alcohol consumption, in diabetic men compared to diabetic women may explain the sex differences in the factors affecting stroke outcomes.

There are several limitations in this study. First, all patients were from a local neurological hospital in Tianjin, China and may not represent all stroke patients in China. This hospital is a specialized neurological hospital located in the center of Tianjin, and a large number of patients were from the city, not from rural areas. Second, the definition of DM was limited to confirm DM before stroke onset, a self-reported medical history of DM, or the use of anti-diabetic medications. This may have resulted in underestimation of the numbers of AIS patients with DM, which would influence the reliability of the study’s conclusions.

## Conclusions

In this large hospital-based stroke registry, there were sex differences in factors associated with outcomes at different stages after stroke. TOAST classification of LAA, CE, and smoking were risk factors of outcomes in women in the short term and medium term; however, AF, obesity, and alcohol consumption were independent predictors of stroke outcomes in men. These findings suggest that it is crucial to establish individualized treatment strategies for every patient according to their different risk factors for stroke, to strengthen stroke rehabilitation, and to provide early health education for the secondary prevention of stroke in patients with DM.
